# Green NADES-Based Pretreatment Combined with Microwave-Assisted Hydrodistillation for Enhanced Fennel Essential Oil Production

**DOI:** 10.3390/molecules30183734

**Published:** 2025-09-14

**Authors:** Songsak Planonth, Aiya Chantarasiri, Jakkrawut Maitip, Nalin Wongkattiya, Sirinat Noyraksa, Suwaporn Luangkamin, Keerati Tanruean, Panawan Suttiarporn

**Affiliations:** 1 Faculty of Science, Energy and Environment, King Mongkut’s University of Technology North Bangkok, Rayong Campus, Rayong 21120, Thailand; songsak.p@sciee.kmutnb.ac.th (S.P.); aiya.c@sciee.kmutnb.ac.th (A.C.); jakkrawut.m@sciee.kmutnb.ac.th (J.M.); 2 Program in Biotechnology, Faculty of Science, Maejo University, Chiang Mai 50290, Thailand; nalin@mju.ac.th; 3 Techno Park, King Mongkut’s University of Technology North Bangkok, Rayong Campus, Rayong 21120, Thailand; sirinat.n@technopark.kmutnb.ac.th; 4 Department of Fundamental Science and Physical Education, Faculty of Science at Sriracha, Kasetsart University, Sriracha Campus, Chonburi 20230, Thailand; suwaporn.l@ku.th; 5 Program in Biology, Faculty of Science and Technology, Pibulsongkram Rajabhat University, Phitsanulok 65000, Thailand; keerati.t@psru.ac.th

**Keywords:** natural deep eutectic solvents, microwave-assisted hydrodistillation, choline chloride, glycerol, pretreatment, fennel, antimicrobial activity, antioxidant activity

## Abstract

Natural deep eutectic solvents (NADESs) are emerging green solvents widely applied to improve the extraction of essential oil (EO) through plant tissue pretreatment. Various NADESs, formulated from polyalcohols, sugars, and organic acids, were employed as pretreatment solvents prior to microwave-assisted hydrodistillation (MAHD) to facilitate plant cell wall breakdown and improve the efficiency of EO extraction. The findings revealed that the most effective pretreatment conditions for enhancing EO extraction involved using a NADES composed of choline chloride and glycerol (in a 1:2 molar ratio), applied to fennel seed powder at a solid-to-NADES ratio of 1:6 g/mL. Optimal performance was achieved with 20% water content in the NADES, microwave irradiation at 400 W for 6 min, followed by 96 min of MAHD. Under these conditions, the NADESs-based MAHD achieved the highest EO yield, increasing it from 1.33% with water-based MAHD to 2.70%. Fennel EO demonstrated the strongest antimicrobial activity against *S. pyogenes* and *C. albicans.*, while the EO obtained from NADES-MAHD using Ch:Gly (1:2) showed the highest antioxidant activity, with 72.41% inhibition. Finally, GC-MS phytochemical analysis of the extracted EOs revealed anethole as the major compound. Notably, the application of NADES, particularly Ch:Gly (1:2), enhanced the relative content of monoterpene hydrocarbons. These findings highlight the superior effectiveness of deep eutectic solvents during the pretreatment stage in enhancing EO production.

## 1. Introduction

**Figure 1:**
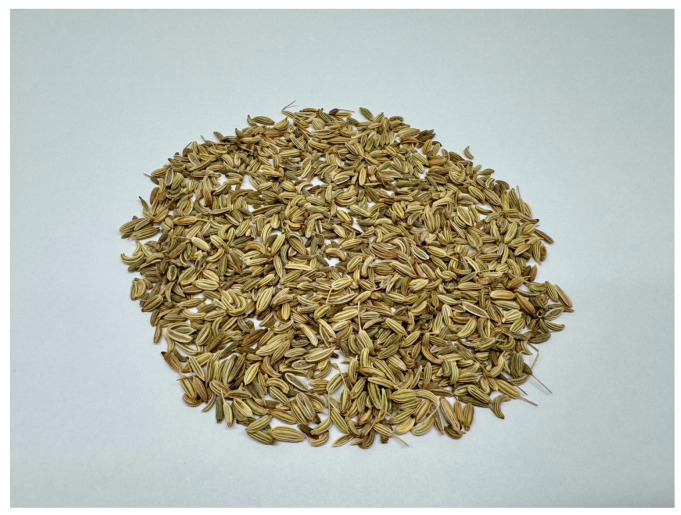
Morphological characteristics of fennel (*Foeniculum vulgare*) seeds.

Fennel (*Foeniculum vulgare*) is a flowering plant originating from the Mediterranean but has become naturalized in many parts of the world [1], as shown in Figure 1. It is widely recognized for its broad range of applications in numerous fields, and traditional medicine frequently prescribes it for kidney stones, vomiting, diarrhea and neurological disorders. In addition to its antiulcer and antiseptic properties, this plant is a good carminative and antispasmodic [2,3,4]. Because fennel seeds contain a number of nutrients, including vitamins and minerals, they are frequently utilized in cooking recipes. It is also used in the culinary, cosmetics, fragrance, and pharmaceutical industries, mostly in beverages and confections [5,6]. In addition, this plant offers added value through the production of essential oils (EOs), which are aromatic compounds extracted and separated from the aqueous phase after distillation.

EOs are concentrated natural oils extracted rich in aromatic compounds, commonly used in aromatherapy for their therapeutic benefits. EOs from *Foeniculum vulgare* exhibit hepatoprotective and antidiabetic effects in vivo models. Owing to their high *trans*-anethole content, these oils demonstrate potent antioxidant activity. Additionally, fennel EOs show antimicrobial effects against Gram-positive and Gram-negative bacteria, as well as fungi, highlighting their potential application in food preservation [7]. Given the diverse benefits of EOs, advancing efficient and sustainable extraction methods has become increasingly important.

EOs are commonly extracted using traditional methods such as hydrodistillation and steam distillation. However, these methods often require long extraction times and yield relatively small amounts of oil, which can lead to the loss of valuable components through hydrolysis or thermal degradation, especially in the case of unsaturated compounds. To address these issues, MAHD has emerged as a green, eco-friendly, and efficient alternative that combines microwave heating with traditional hydrodistillation principles. The development and adoption of MAHD EO extraction is highly desirable. Due to its advantages including shorter extraction time, faster heating, improved product quality, energy efficiency, and no direct contact between the plant and heat source, MAHD is widely used for extracting volatile compounds from various plants [8].

Additionally, pretreatment is essential for overcoming the natural resistance of plant cell walls and enhancing the release of intracellular bioactive compounds. Recently, deep eutectic solvents (DESs) have emerged as a novel class of green solvents offering a promising alternative for such applications. They are typically made by mixing hydrogen bond donors (HBD) and hydrogen bonds acceptors (HBA) in various molar ratios; this results in a combination that has a lower melting point because of the hydrogen bonding. Low toxicity, affordability, biodegradability, broad liquid range, low vapor pressure, non-flammability, chemical and thermal stability, ease of synthesis with excellent purity, water compatibility, and strong solubilizing power are only a few benefits of DESs. DESs made from solely natural substances are termed natural deep eutectic solvents (NADESs) [9]. NADESs have emerged as versatile green solvents, enabling the efficient extraction of diverse bioactive compounds from plants through a variety of advanced extraction techniques. Antioxidant compounds from Thai pigmented rice bran [10], Anthocyanins from chokeberry fruit [11] and from black raspberry [12], hydrophilic and lipophilic compounds from brown seaweeds [13], and coumarins from *Angelicae pubescentis* [14] were obtained by ultrasonically assisted extraction (UAE) with the assistance of NADES. Curcuminoids were reported to be extracted from turmeric by heating turmeric powder in the presence of NADES [15]. NADES were demonstrated as promising green solvents that can efficiently extract triterpene saponins from *Aralia elata* roots, in some cases outperforming conventional alcohol-based methods [16]. Microwave-assisted hydrodistillation (MAHD) is an advanced technique that overcomes limitations of traditional methods by enabling faster and more efficient extraction of essential oils from diverse plant materials. MAHD utilizes microwave radiation to heat the water within plant cells, leading to evaporation and expansion that generate high internal pressure. This pressure stretches and may rupture the oil gland cell walls, thereby facilitating the release of essential oils or bioactive compounds. The process benefits from a synergistic effect of heat and mass transfer, which work together to enhance extraction efficiency. Compared to conventional methods, MAHD is a green and innovative extraction technique that offers rapid extraction, higher essential oil quality and yield, energy efficiency, cost-effectiveness, and minimal solvent usage, thereby reducing both cost and environmental impact [17]. However, the technique also has limitations, particularly the risk of thermal degradation of thermo-sensitive compounds under excessive microwave power or prolonged heating that influence yield and product quality [18]. NADESs have been mostly used with microwave-assisted extraction method [19]. Rapid heating by microwave together with the advantages of NADES make this combination attractive. The microwave hydrodistillation based on deep eutectic solvent has been applied in the extraction of EOs from Amomum Species [20], *Liquidambar formosana* leaves and fruits [21], and turmeric [22]. In addition, we previously reported the use of DES-based microwave-assisted hydrodistillation (DES-MHD) and ultrasonic-assisted DES pretreatment followed by microwave-assisted hydrodistillation (U-DES-MHD) of clove [23].

Despite the recognized potential of EO extraction from *F. vulgare*, studies focusing on green pretreatment techniques to enhance EO yield are still limited to only a few reports. To address this research gap, the objective of this study is to evaluate a novel green extraction method, employing NADES-based pretreatment with microwave-assisted hydrodistillation (MAHD) for the efficient recovery of EOs from *F. vulgare* seeds. The investigation will focus on varying parameters in the pretreatment stage. Those parameters include the type of NADES, solid-to-NADES ratio, water content, microwave power, and pretreatment duration. Furthermore, the extracted EOs were evaluated for their antimicrobial and antioxidant activities, along with an analysis of their phytochemical compositions.

## 2. Results

### 2.1. Effects of NADES Components

**Figure 2:**
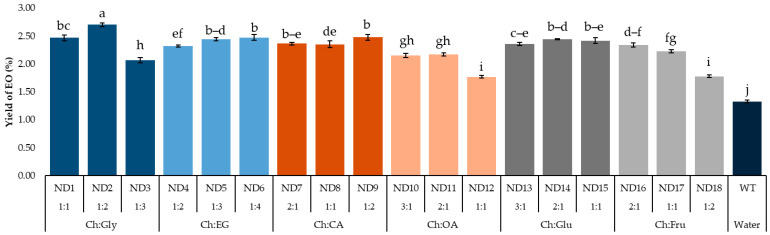
EO yield from *F. vulgare* with different NADESs. Data are presented as mean ± standard deviation (SD) from n = 3 independent experiments. Abbreviations are as follows—Ch: Choline chloride; Gly: Glycerol; EG: Ethylene glycol; CA: Citric acid; OA: Oxalic acid; Glu: Glucose; Fru: Fructose; EOs: Essential oils. Different lowercase letters indicate significant differences among groups based on Tukey’s pairwise comparison test (*p <* 0.05); bars sharing the same letter are not significantly different, while bars with different letters are significantly different in the measured response.

Efficiency of NADES-MAHD using different types and molar ratios of NADESs represented as EO extraction yields shown in Figure 2. When microwave irradiation is used in an extraction, the dipole moment of the medium directly affects the extraction efficiency. This is because polar molecules can absorb microwave irradiation, which results in fast heating and breaking of cell structure. This phenomenon results in releasing compounds from the cell [24,25]. Furthermore, variations in the chemical structures of DESs affect EO yields due to their differing polarities and cellulose-dissolving capabilities [26].

EO yields were in the range of 1.33–2.70%. The highest yield was obtained using Ch:Gly at a molar ratio of 1:2 (ND2) as NADES. Therefore, it was selected for further investigation. It is worth mentioning that, although not giving the highest extraction yield, NADES composed of Ch:EG, Ch:CA and Ch:Glu provided remarkable high yield at 2.32–2.48% for all ratios in this study.

Due to the similar dipole moments of glycerol and ethylene glycol, NADESs based on Ch:Gly and Ch:EG showed comparable extraction yields. For carboxylic acids, an overall yield of EOs from Ch:CA was found to be higher than that of Ch:OA. This effect can be attributed to the intrinsic properties of citric acid, which exhibits a relatively high dipole moment and an abundance of hydroxyl and carboxyl groups, thereby promoting extensive hydrogen-bonding interactions that enhance the structural stability of the system [27].

Glucose was reported slightly higher dipole moment than fructose. This is due to the different ring size and arrangement of hydroxyl groups. As a result, when used as NADESs in NADES-MAHD, Ch:Glu represented a higher average yield of EOs than that from Ch:Fru. The polarity of DESs varies depending on the hydrogen bond donor (HBD) component used. Organic acid-based DESs are generally the most polar, while sugar- and polyalcohol-based DESs exhibit lower polarity, similar to that of methanol. Additionally, the polarity of a NADES can be fine-tuned by adjusting the molar ratio of its components. Since solvent polarity strongly influences the extraction efficiency of EO compounds, matching the polarity of the NADES with the oil composition typically leads to higher yields [28].

All types of NADES-based MAHD resulted in higher EO yields (1.77–2.70%.) compared to water-based MAHD without pretreatment (1.33%), indicating that the use of NADESs as a pretreatment for turmeric had a significant impact on enhancing EO yield. These interactions increase cell wall permeability, thereby facilitating EO extraction. As NADES are composed of natural plant-compatible components, they represent a promising green solvent for improving EO isolation [29].

Although Ch:Gly at a 1:2 molar ratio (ND2) provided the highest yield (2.70%), increasing the ratio to 1:3 (ND3) led to a significant decline (2.07%). Similar trends were observed for Ch:OA (ND12), with a yield of 1.77%, and Ch:Fru (ND18), with a yield of 1.78%. This reduction is likely due to the decreased amount of choline chloride, which lowered the proportion of hydrogen bond acceptors in the NADES system. Since hydrogen bonding is crucial for solubilizing bioactive compounds, especially those with polar functional groups like hydroxyls, this imbalance may impair extraction efficiency. These findings highlight the importance of maintaining an optimal donor-to-acceptor ratio in NADES formulations [30].

### 2.2. Effects of Solid-to-NADES Ratio

**Figure 3:**
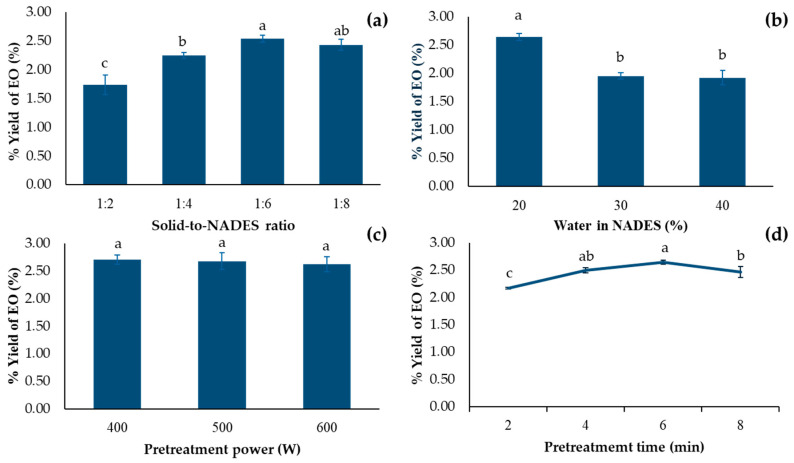
EO yield from *F. vulgare* with different (**a**) amount NADESs (**b**) water content in NADES (**c**) pretreatment power and (**d**) pretreatment time. Abbreviations are as follows—NADES: Natural deep eutectic solvent; EOs: Essential oils. Data are presented as mean ± standard deviation (SD) from n = 3 independent experiments. Different lowercase letters indicate significant differences among groups based on Tukey’s pairwise comparison test (*p <* 0.05); bars sharing the same letter are not significantly different, while bars with different letters are significantly different in the measured response.

Solid-to-NADES (S/N) ratio is one of the most important parameters in the pretreatment step of EO extraction, as shown in Figure 3a. With the small amount of NADES, i.e., high S/N ratio (1:2 and 1:4), low EO yield were obtained. The reason behind this was the saturation of EO in solvent, which led to an incomplete extraction. In addition, too little amount of solvent could lead to a complete evaporation, which resulted in burning of the seeds [31]. The higher yield was obtained when increasing quantity of NADES-lowering S/N ratio. This is probably because of a larger concentration gradient of EO between matrix and the solvent [32]. However, too low S/N ratio (1:8) resulted in dropping of extracted yield. This is because, with a low S/N ratio, longer time is required due to high dilution [31]. With pretreatment in this condition, it was possible that the pretreatment step was not completed. Therefore, the optimal S/N ratio for NADES-MAHD of EO from fennel seed was 1:6 g/mL.

### 2.3. Effect of Water Content in NADES

Water plays a major role in NADES properties. High viscosity is one of the distinct characteristics of NADES due to the extensive hydrogen bonding. Adding water to NADES decreased this viscosity, making solvent molecules easier to contact fennel seeds [33]. However, excessive water results in decreasing interaction between NADES and targeted molecules [24].

A water content of 20–30% in NADES is generally recommended, as it can enhance the extraction efficiency of both polar and non-polar compounds [30]. However, the optimal water level should be tailored to suit each specific application. With 3 different levels of water in NADES (20%, 30% and 40%), the 20% formulation provided the highest extraction yield (2.65%), as shown in Figure 3b.

In addition to decreasing viscosity, water in NADES also affected the hydrogen-bond network of NADES, a phenomenon that disrupts the plant cell wall or improves the solubility of EOs. With too high water content, NADES loses this network, hence the decreased extracted yield [34,35]. Different water content also alternated NADES polarity. Higher water content made NADES more polar, which was undesired for the extraction of non-polar EOs.

Recent studies have shown that water content influences not only the viscosity of lactic acid-based NADES but also several physicochemical and antimicrobial properties. Dilution with water weakens hydrogen bonding, decreases density and refractive index, increases pH, and significantly reduces viscosity and surface tension. Moreover, higher water content increases water activity and leads to a reduction in antimicrobial activity. These findings highlight the importance of controlling water content when designing NADES for green extraction and antimicrobial applications [36].

### 2.4. Effect of Pretreatment Power

Microwave power was suspected to improve extraction efficiency by inducing molecular vibrations that disrupt plant cell walls during the pretreatment stage, thereby facilitating dissolution and allowing EOs to diffuse out [26]. Previous study found the obvious different extracted yield when using different microwave power in MAHD step [37]. However, as shown in Figure 3c, no significant difference in EO yield was observed when the microwave power was increased from 400 to 600 W, with yields ranging from 2.65% to 2.71%. Therefore, 400 W was the optimum microwave power for this step.

### 2.5. Effect of Pretreatment Time

The extraction procedure consisted of two steps: NADES pretreatment and MAHD. Besides NADES compositions, parameters including pretreatment duration were investigated. Pretreatment time directly affected the yield of EO. As shown in Figure 3d, increasing the pretreatment time from 2 to 6 min resulted in an increase in EO recovery yield from 2.17% to 2.65%. Prolonging the pretreatment to 8 min resulted in a reduced recovery yield of 2.47%, which may be attributed to the thermal degradation of the EOs [38]. Considering power consumption and the lack of significant difference between pretreatment durations of 4 min and 6 min, the 4 min pretreatment was identified as the most suitable option.

### 2.6. Antimicrobial Activities

All fennel EOs were examined for their antimicrobial activity against four Gram-positive and four Gram-negative bacteria, as outlined in Table 1. In comparison to the antibiotic tetracycline, which served as positive control, all EOs exhibited significantly lower antimicrobial activities. Notably, every EOs extracted using MAHD, including the sample that did not undergo NADES pretreatment, demonstrated the strongest antimicrobial activity against *S. pyogenes*. Specifically, the EOs extracted with Ch:Gly (1:2) and Ch:CA (1:2) were particularly effective, showing the most potent antibacterial effects. Additionally, all EOs were active against *L. monocytogenes* and *Salmonella Typhi*. However, none of the EOs demonstrated the ability to inhibit *P. aeruginosa*. These findings indicate that NADESs did not alter the antimicrobial activity of the EOs. Previous studies have described fennel oil as being antimicrobial against *S. aureus* and *E. coli* [39,40]. Although Naaz et al. reported that fennel oil inhibited *P. aeruginosa*, this activity was not observed in the current study [40]. Minimum inhibitory concentration (MIC) of EOs ranged from 1.56 to 25 mg/mL (Table 2). EO extracted with Ch:CA (1:2) pretreatment seems to be the most active against bacteria. All EOs were found to be most active against *Str. Pyogenes* and least active against *P. aeruginosa.* These results correspond to an agar disk diffusion experiment.

The antifungal activities of fennel EO extract are detailed in Table 1. For each type of NADES, only the EO with the highest extraction yield was used to evaluate antifungal activity. The most effective extract against *C. albicans* was obtained from MAHD without NADES pretreatment. Although NADES pretreatment resulted in slightly lower antifungal inhibition compared to MAHD alone, it still offered advantages in extraction efficiency. Notably, EOs obtained using NADESs maintained antifungal activity that exceeded that of the positive control, nystatin. Among the NADES extracts, Ch:Fru (2:1) exhibited the highest inhibition activity at 11.40 ± 0.27 mm, followed closely by Ch:CA (1:2) at 11.35 ± 0.25 mm, and Ch:Gly (1:2) at 10.68 ± 0.15 mm. These findings align with previous research [41], which also demonstrated the antifungal properties of fennel oil against *C. albicans*. EOs are recognized for their effective antifungal activity against *C. albicans* and show great potential for future medical applications due to their low toxicity and multifunctional properties [42].

In addition to their antimicrobial properties, it is important to consider the cytotoxic potential of fennel essential oil. Sharopov et al. (2017) reported that the essential oil of fennel seeds exhibits low cytotoxicity against various cancer cell lines, including HeLa (human cervical cancer), Caco-2 (human colorectal adenocarcinoma), MCF-7 (human breast adenocarcinoma), CCRF-CEM (human T lymphoblast leukemia), and CEM/ADR5000 (adriamycin-resistant leukemia), compared to the chemotherapy drug doxorubicin [43].

### 2.7. Antioxidant Activity

Antioxidant capacity of EOs is shown in Table 3. It appears that, besides elevating extraction yield, NADES also affects the activity of the resulting EOs. While most of the EOs exhibited inhibition in the same range as that from MAHD without NADES, the EO obtained from NADES-MAHD using Ch:Gly (1:2) as NADES showed a higher activity, with 72.41% inhibition and an IC_50_ value of 8.03 ± 0.16 mg/mL. In contrast, when NADES was Ch:CA (1:2), the resulting EO had less activity, showing only 60.81% inhibition. Effects of NADES on EOs’ antioxidant capacity were not surprising as we previously found that NADES affected DPPH radical scavenging activity of EO from clove [23]. Nevertheless, the fennel seed EO exhibited lower antioxidant activity compared with the positive control, BHT, which showed an IC_50_ of 8.05 ± 0.95 µg/mL. However, according to [44], fennel EOs exhibited notable antioxidant activity, with IC_50_ values ranging from 11.83 to 36.90 mg/mL in the DPPH assay. The radical scavenging capacity of the EOs may be attributed to certain compounds that are capable of donating a hydrogen atom to the DPPH^•^ free radical, thereby converting it to the reduced form, DPPH-H [44].

Nevertheless, previous studies have suggested that compounds such as fenchone and anethole contribute to the phenolic content and antioxidant activity of fennel, while monoterpene hydrocarbons (e.g., those present in thyme and oregano) play a significant role in inhibiting oxidation–peroxidation [45]. Beyond the DPPH assay used in the present work, other evaluations have also demonstrated the antioxidant potential of fennel seed EO, including its inhibitory efficacy against lipid peroxidation and remarkable Fe-binding capacity, indicative of metal-chelating activity. Collectively, these findings reinforce the role of fennel EO as both a free radical scavenger and an oxidation protector through multiple mechanisms [40].

### 2.8. Volatile Phytochemical Compositions of Fennel Seed EOs

**Figure 4:**
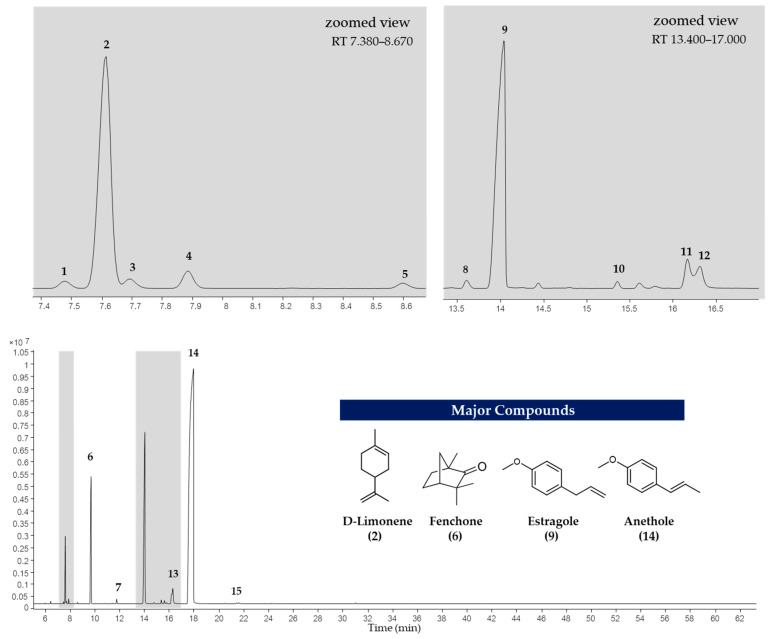
Total ion chromatogram of EO extracted using NADES-MAHD.

**Figure 5:**
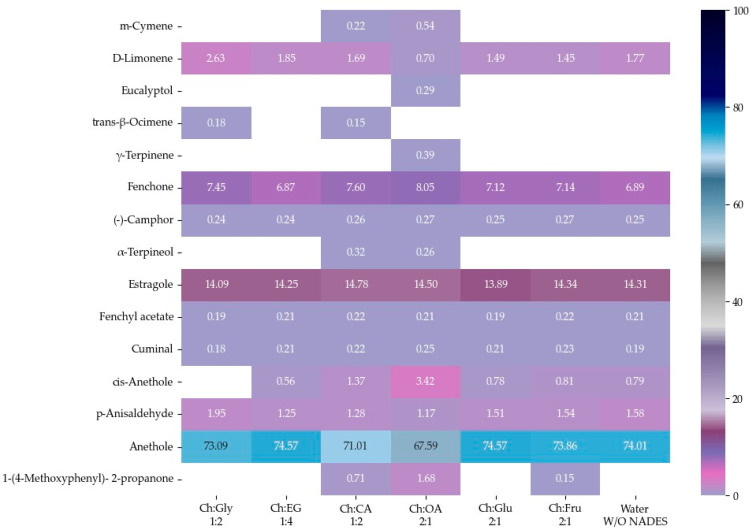
Heat map of relative area percentage of volatile phytochemical compositions in EOs.

**Figure 6:**
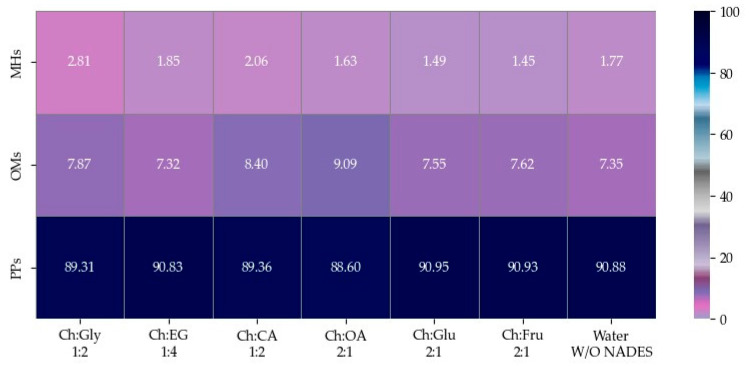
Heat map of relative area percentages of phytochemical classes in EOs.

The EOs of *F. vulgare* extracted using each type of NADES, as well as those extracted with water (without NADES), were analyzed for their chemical constituents using GC-MS. The total ion chromatogram is shown in Figure 4, and the results are summarized in Figure 5 and Figure 6. Based on the data (Table 4), a total of 15 volatile compounds were identified and quantified across the three groups, including 4 monoterpene hydrocarbons (MHs), 5 oxygenated monoterpenes (OMs), and 6 phenylpropanoids (PPs), as shown in Figure 6. Of all EOs, the major compounds were phenylpropanoids (PPs), specifically anethole and estragole. It is worth noting that all EOs consisted of anethole at the minimum level of 71.01% except that obtained by using Ch:OA as NADES, in which anethole was only 67.59%.

In addition, oxygen atoms in fenchone probably made the difference by interacting with NADES, making the compound more accessible to solvents. EO obtained by Ch:CA (1:2) and Ch:OA (2:1) also demonstrated other interesting constituents besides a higher number of compounds compared to other EOs.

The 4 major compounds (anethole, estragole, fenchone and D-limonene) extracted by MAHD, without any NADES, were found identical to those reported by Diao et al. [46], in which fennel seeds were extracted by conventional hydrodistillation (HD). Diao et al. had found that D-limonene was slightly higher than fenchone (5.45% to 6.24%) while this study found fenchone (6.87% to 8.05%) [46] much higher than D-limonene (0.70–2.63%). It is proposed that two reasons behind this phenomenon are that fenchone possesses a higher dipole moment than D-limonene and therefore interacts more effectively with microwaves.

Considering, α-terpineol was a compound found only when carboxylic acids were used as HBDs, Ch:OA (2:1). *trans*-β-Ocimene was found in EOs extracted using Ch:Gly 1:2 and ChCA (1:2). 1-(4-Methoxyphenyl)-2-propanone was observed in EOs obtained from systems using carboxylic acids as HBDs as well as Ch:Fru (2:1). The EO extracted using Ch:Gly (1:2) contained the highest levels of monoterpenes, particularly D-limonene and *trans*-β-ocimene, highlighting its remarkable efficiency in capturing key aromatic compounds. Consistent with [20], the application of DES led to an increase in the relative content of hydrocarbons in most EOs, indicating that the enhanced EO yield resulted primarily from the release of more intracellular bioactive compounds rather than from oxidation of EO components.

Multiple studies have demonstrated the antimicrobial effects and underlying mechanisms of D-limonene against a range of bacterial and fungal species, while β-ocimene, a key component in EOs, has been associated with anticonvulsant, antifungal, and antitumor activities, along with pest-resistant properties [47]. These observations indicate that suitable NADES can enhance the extraction of target compounds. Their superior performance may result from stronger hydrogen bonding or specific interactions with analytes, improving solubility and diffusion. Thus, tailoring NADES compositions to match compound structures can boost yield and selectivity, promoting greener extraction technologies [34].

## 3. Discussion

In recent years, growing environmental awareness has led companies to adjust their ethical standards and production practices. The drive toward global sustainability and the concept of a “green industry” calls for practical solutions that reduce environmental impact while preserving product quality [48]. This study successfully developed an innovative and eco-friendly extraction strategy using NADES pretreatment to significantly enhance the efficiency of MAHD for isolating essential oils from *Foeniculum vulgare*. These green NADESs facilitate cell wall disruption and enhance the release of volatile compounds, resulting in a substantially higher yield. NADES are natural solvents composed of primary or secondary metabolites mainly derived from living organisms, especially plants, such as sugars, amino acids, organic acids, fatty acids, or chlorine derivatives. Due to their natural origin, these solvents exhibit low toxicity, high biodegradability, and stability when mixed. Such properties confirm the suitability of NADES as environmentally friendly solvents and highlight their potential as a promising and sustainable alternative to conventional extraction methods for the valorization of plant materials [49]. One of the most remarkable features of NADES is their high tunability, which allows their properties to be tailored by selecting appropriate components and molar ratios. This tunability affects both physical properties (e.g., viscosity, pH) and chemical characteristics (e.g., polarity), enabling their customization for specific applications [34]. In hydrophilic DES systems, the choice of HBD plays a key role in determining viscosity. Systems containing phenol, glycols, or ethylene glycol generally exhibit reduced viscosity, whereas those composed of choline chloride with urea, polycarboxylic acids, or sugars tend to have medium to high viscosity [50]. For DESs containing 30% water at a 1:1 HBA:HBD ratio, the viscosity follows the order: sugar-based DES > glycerol-based DES > organic acid-based DES > ethylene glycol-based DES > amide-based DES. Correspondingly, the pH values vary, with organic acid-based DESs showing the lowest pH (<1), followed by sugar-based (3.39–4.15), alcohol-based (4.91–5.37), and amide-based DESs (7.08–7.35). Such tunability not only governs their solvent characteristics but also enables interactions with specific compounds, including essential oil constituents, through non-covalent forces such as hydrogen bonding and electrostatic (both repulsive and attractive) interactions [10].

The higher extraction yield observed in NADES-based MAHD compared to water-based MAHD can be attributed to the superior penetration capacity of NADES constituents. These components absorb microwave radiation more effectively than water and convert it into thermal energy, which facilitates the dissolution of cell wall components and the extraction of soluble substances from the plant material. This enhanced performance is largely due to the unique role of hydrogen bond donors (HBD) under microwave irradiation, which promotes compound release through efficient microwave absorption, disruption of the cell wall, and cellulose dissolution [51,52].

The integration of NADES with microwave-assisted hydrodistillation (NADES-MAHD) not only enhances essential oil yield but also significantly improves energy efficiency compared to conventional hydrodistillation (HD). By reducing extraction time and lowering electricity consumption, NADES-MAHD minimizes the overall energy demand of the process. For instance, during the extraction of essential oils from *Litsea cubeba* fruits, conventional HD required 0.76 kWh, while MAHD and NADES-MAHD consumed only 0.40 and 0.39 kWh, respectively. This substantial reduction highlights the benefit of combining green solvents with microwave technology, as it translates into lower operational costs, reduced carbon emissions, and improved sustainability. Thus, NADES-MAHD offers a promising pathway for scalable essential oil production with minimized environmental impact [24].

Interest in essential oils has steadily increased in recent years due to their high economic value and expanding market demand. These aromatic compounds are widely applied in the food, cosmetics, and pharmaceutical industries. Despite their potential, scaling up essential oil extraction using microwave-assisted hydrodistillation (MAHD) remains challenging, primarily because of the limited penetration of microwaves into plant materials [53]. Nevertheless, the study by Lamberti et al. demonstrated the feasibility of scaling MAHD from laboratory to pilot scale. Using the ETHOS XL system, they obtained yields comparable to those achieved at the laboratory scale while processing more than six times the biomass within the same time frame. This significantly enhanced productivity, with yields from pellets increasing nearly fourfold and those from dry cones almost doubling, underscoring the strong potential of MAHD for industrial application, particularly given the industry’s preference for dried and pelletized hops [54].

Beyond extraction efficiency, an important consideration for the industrial application of NADES is their toxicological safety, particularly the potential risk of co-extracting toxic trace elements from plant matrices. Although NADES are composed of generally recognized as safe (GRAS) components, their strong solubilizing power may lead to the unintended extraction of metals or other impurities. This concern has been recently addressed in a study on *Glycyrrhiza glabra* roots, where acid-based NADES were shown to co-extract glycyrrhizic acid along with trace elements. Interestingly, the recovery of all trace elements (except Li) was relatively low (<6%), and statistical analysis indicated that the hydrogen bond donor type was the decisive factor influencing element extraction. More importantly, comprehensive health risk assessments, including the metal pollution index, hazard quotient, hazard index, and chronic daily intake, confirmed that all tested NADES extracts were nontoxic and posed no health risk for either ingestion or topical application [55].

## 4. Materials and Methods

### 4.1. Materials and Chemicals

Choline chloride (98%) was purchased from Loba Chemie Pvt Ltd., Mumbai, India. Oxalic acid dihydrate (≥99.5%) was purchased from Qrec (Asia), Rawang, Malaysia. Glycerol (≥99.5%), ethylene glycol (≥99.9%), citric acid 1-hydrate (99.5–100.5%), D-glucose-hydrate (97.5–102%) and D-fructose (98.0–102.0%) were purchased from Elago Enterprises Pty Ltd., Cherrybrook, Australia. 2-Diphenyl-1-picrylhydrazyl (DPPH) and alkane standard solution were bought from Sigma Aldrich Chemical Co. (St Louis, MO, USA). The compound 2,6-di-tert-butyl-4-methylphenol (BHT) was provided from Acros, Dreieich, Germany. Yeast malt broth and mueller hinton broth were from HiMedia, Kennett Square, PA, USA, while agar powder was from Krungthepchemical, Bangkok, Thailand. The material was authenticated by the Faculty of Science and Technology, Pibulsongkram Rajabhat University (PSRU), Phitsanulok, Thailand. *Foeniculum vulgare* Mill. (Fennel) was preserved with a specimen number (PSRU1233). Fennel seeds were oven-dried at 40 °C until a constant weight was achieved. Dried seeds were grounded and filtered through a 250-µm sieve.

### 4.2. Preparation of NADESs

The selection of hydrogen bond donors (HBDs) was guided by previous studies demonstrating their effectiveness in forming NADES with choline chloride for essential oil extraction. Ethylene glycol, glycerol, oxalic acid, citric acid, glucose, and fructose were selected as hydrogen bond donors (HBD) along with choline chloride (HBA) as NADESs because previous research utilized ethylene glycol, glycerol, oxalic acid, and fructose along with choline chloride as a DES-based microwave-assisted hydrodistillation method (DES-MAHD) to extract clove essential oils [23], while other studies employed oxalic acid, citric acid, and glucose along with choline chloride as a DES-based MAHD to extract essential oils from fruits of *Litsea cubeba* (Lour.) [24].

Both glucose and fructose were selected as HBDs along with choline chloride (HBA) due to previous findings in the literature. Yu et al. (2017) employed glucose, fructose, ethylene glycol, glycerol, and urea as HBDs with choline chloride to extract essential oils from pepper fruits using the DES-based MAHD method and observed that fructose as HBD gave the highest essential oil yield [56]. In contrast, Xu et al. (2021) used glucose, fructose, and oxalic acid as HBDs with choline chloride to extract turmeric oils by the DES-based MAHD method, finding that glucose provided a higher yield than fructose [22]. Based on these studies, choline chloride (Ch) was selected as the sole HBA in our work, while the HBDs were varied as glycerol (Gly), ethylene glycol (EG), citric acid (CA), oxalic acid (OA), glucose (Glu), and fructose (Fru) to investigate their effects on essential oil extraction. The HBA and HBD were mixed at varying molar ratios, as summarized in Table 5. Each mixture was heated with magnetic agitation until clear liquid was formed.

### 4.3. NADES-MAHD for EO Extraction

**Figure 7:**
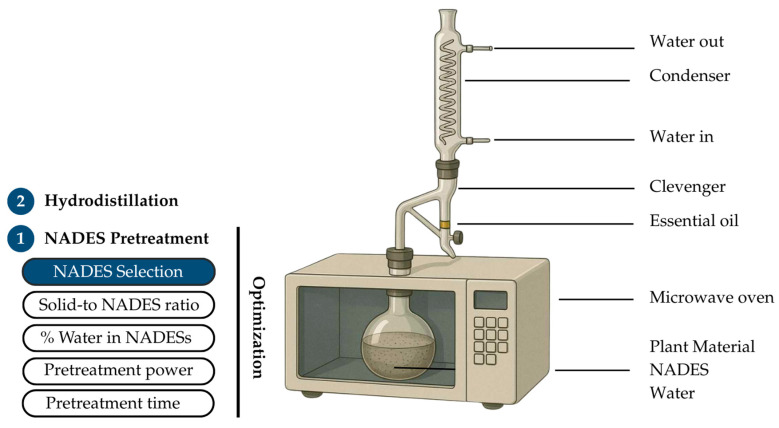
Overview of the NADES-MAHD extraction process, highlighting 2 major steps and key pretreatment optimization parameters.

Microwave-assisted natural deep eutectic solvent pretreatment coupled with hydrodistillation (NADES-MAHD) was selected as the extraction method. The schematic diagram of the extraction setup is shown in Figure 7. The apparatus consisted of a microwave oven (TOSHIBA ER-SGS34(S)TH 34, Toshiba Thailand Co., Ltd., Bangkok, Thailand), connected to a Clevenger-type apparatus for the collection of EOs. This configuration allowed for efficient energy transfer during pretreatment and facilitated the separation and condensation of volatile compounds during the hydrodistillation step. The process consisted of 2 main steps: pretreatment and hydrodistillation. In step 1, the pretreatment stage using NADESs facilitated the release of EO components from the fennel seed matrix, enabling step 2, hydrodistillation, to effectively separate the EOs from the extraction medium. During the pretreatment step, key parameters, including the type of NADES, solid-to-NADES ratio, water content in the NADES, pretreatment time, and microwave power, were systematically optimized. NADESs with 20% water content was mixed with grounded fennel seed at the solid-to-NADES ratio of 1:6 (g/mL). The mixture was heated with microwave irradiation at 400 W for 4 min. During hydrodistillation, 270 mL of deionized water was added to the mixture, followed by microwave heating at 400 W for 96 min, based on the optimized MAHD conditions previously reported by our group [37]. After hydrodistillation, the fennel EO was separated from aqueous phase and was weighed. The yield of EOs in each extraction condition was calculated according to Equation (1)

Yield of EO(%) = W_E_/W_F_ × 100% (1)

where W_E_ is the weight of fennel EO, and W_F_ is the weight of fennel seed powder.

### 4.4. Antimicrobial Assay

The antimicrobial activities of fennel EO extract were evaluated using the agar disk-diffusion method. A suspension containing a final inoculation size of 0.5 McFarland was prepared for four Gram-positive bacteria (*Staphylococcus aureus* DMST 8840, *Streptococcus pyogenes* DMST 30563, *Bacillus cereus* DMST 5040, and *Listeria monocytogenes* DMST 17303) and four Gram-negative bacteria (*Escherichia coli* DMST 4212, *Salmonella Typhi* DMST 5784, *Pseudomonas aeruginosa* DMST 4739, and *Enterobacter aerogenes* DMST 8841). These suspensions were spread-plated on Mueller Hinton Agar (MHA) (HiMedia, Thane, India). For each test bacterium, a 6.0 mm diameter filter paper disk was placed on the agar surface, and 10 µL of EO was added. Additionally, tetracycline (Oxoid, Ogdensburg, NY, USA) at a concentration of 30 µg was used as a positive control. The agar plates were incubated at 37 °C for 24 h, and the sizes of the inhibition zones were measured. All experiments were conducted in triplicate.

A suspension of *Candida albicans* was prepared to reach a final inoculation size of 0.5 McFarland and was then spread-plated on Potato Dextrose Agar (PDA) (HiMedia, Thane, India). For each EO tested, a 6.0 mm diameter filter paper disk was placed on the surface of the agar, and 15 µL of the EO was applied to the disk. The agar plates were incubated at 25 °C for 48 h, and the size of the inhibition zone was measured afterward. Nystatin (Alfa Aesar, Lancashire, UK) at a concentration of 50 µg served as the positive control. All experiments were performed in triplicate.

Minimum inhibitory concentrations (MIC) were determined using broth dilution method in 96-well plates. A series of two-fold dilutions of each EO, ranging from 25 to 0.01 mg/mL, was prepared in MHB with the volume of 50 µL. The solution was then added 50 µL of bacteria culture containing approximately 1.5 *×* 10^6^ CFU/mL. The plates were incubated at 37 °C for 24 h. The MICs were determined as the lowest concentration of EO inhibiting visible growth of each organism. Tetracycline was used as a positive control, and the experiment was carried out in triplicate.

### 4.5. DPPH Radical Scavenging Assay

A 50 μL aliquot of EO stock solution (diluted 5-fold) was mixed with 150 μL of DPPH solution (0.417 mM). Methanol was used as the blank. After incubation for 30 min at room temperature in the dark, the absorbance was measured at 517 nm using a microplate reader (Metertech, Taipei, Taiwan). The percentage of DPPH radical inhibition was calculated using Equation (2), as follows:

% DPPH radical scavenging = (A_control_ − A_sample_)/A_control_ × 100 (2)

where A_control_ was the absorbance of the control and A_sample_ was the absorbance of the EO solution.

### 4.6. GC–MS-Based Analysis of Fennel Seed Phytochemicals

EO samples were diluted with dichloromethane, and anhydrous sodium sulfate was added to eliminate moisture from the solution. The phytochemical compositions in the EOs were identified and quantified by gas chromatography–mass spectrometry (GC–MS) using an Agilent 6890N GC coupled with a 5973 MSD (Agilent Technologies, Santa Clara, CA, USA), equipped with an HP-5MS capillary column (30 m × 0.25 mm i.d., 0.25 µm film thickness). Helium was used as the carrier gas at a constant flow rate of 1.0 mL/min. Sample injection was performed in split mode with a split ratio of 20:1, using an injection volume of 1 μL and an injector temperature of 230 °C. The column oven temperature was initially set at 60 °C and then ramped to 250 °C at a rate of 3 °C/min. Mass spectrometric detection was conducted using electron ionization (EI) at 70 eV, with a scan range of *m/z*. The ion source and quadrupole temperatures were maintained at 230 °C and 150 °C, respectively.

Compound identification in fennel seed EO was carried out by matching the acquired mass spectra with those available in the NIST Mass Spectral Search Program database. Additionally, confirmation was supported by comparing the Kovats retention indices (RIs), calculated based on the retention times of the target compounds relative to a homologous series of n-alkanes (C_9_–C_26_), with reference values reported in the literature and included in the NIST database. A compound was considered positively identified when both the mass spectral data and RI showed strong agreement. Quantification was achieved by applying the peak area normalization method.

### 4.7. Statistica Analysis

All experimental data are expressed as mean ± standard deviation. Data analysis was performed using the trial version of Minitab 18 software, including analysis of variance (ANOVA) and Tukey’s post hoc test to determine statistically significant differences among means (*p* < 0.05).

## 5. Conclusions

This study successfully developed a novel green extraction strategy combining NADES pretreatment with MAHD to isolate essential oils from *Foeniculum vulgare*. By employing NADES of natural origin, characterized by low toxicity and high biodegradability, the method enhances extraction efficiency while reinforcing its role as a sustainable alternative to conventional techniques. The highest EO yield was achieved using Ch:Gly (1:2 molar ratio) under optimal conditions, including a solid-to-NADES ratio of 1:6 g/mL, 20% water content, microwave power of 400 W, and 6 min of microwave duration, resulting in a significantly higher EO yield compared to conventional water-based MAHD. NADESs are recognized as effective pretreatment solvents owing to their remarkable ability to disrupt cellulose, hemicellulose, and lignin structures. The EO exhibited antimicrobial activity against *S. pyogenes* and *C. albicans*, as well as DPPH radical scavenging activity.

Although the overall EO composition remained consistent, NADES pretreatment led to a higher content of monoterpene hydrocarbons, with D-limonene and *trans*-β-ocimene uniquely detected in the extract obtained using Ch:Gly (1:2). The increased hydrocarbon content and EO yield observed with this NADES formulation could be attributed to its cellulose-dissolving capability, which facilitated greater release of intracellular compounds. The developed method offers an environmentally friendly strategy for producing fennel EO with a targeted composition. NADESs, as tailor-made solvents, possess excellent physicochemical properties that can be fine-tuned to selectively enhance the extraction of specific compounds, allowing precise control over EO profiles. In addition, NADES extracts are non-toxic and are not expected to pose any health risks from either topical application or ingestion, further supporting their potential as safe and sustainable solvents for greener and more efficient pretreatment processes, particularly in the food industry.

Despite the promising results of NADES-assisted MAHD for essential oil extraction, comprehensive optimization of process parameters is still required to fully enhance extraction efficiency and reproducibility. In addition, further studies are needed to assess the stability of essential oil components in NADES and to evaluate the feasibility of solvent reuse. Addressing these challenges will be crucial for meeting the stringent quality standards of the pharmaceutical, cosmetic, and food industries, and for supporting the industrial-scale adoption of NADES-MAHD as a sustainable extraction technology.

## Figures and Tables

**Table 1:** Agar disk diffusion of EOs extracted by NADES-MAHD and without NADES.

Title 1 Microorganism	Inhibition Zone (mm)							
	Positive Control	Ch:Gly 1:2	Ch:EG 1:4	Ch:CA 1:2	Ch:OA 2:1	Ch:Glu 2:1	Ch:Fru 2:1	Water (W/O NADES)
Bacteria								
Gram +								
*S. aureus*	27.00 ± 1.0 ^a^	8.33 ± 0.58 ^b^	8.00 ^b^	8.33 ± 0.58 ^b^	8.00 ^b^	8.33 ± 0.58 ^b^	8.00 ^b^	8.00 ^b^
*S. pyogenes*	38.67 ± 0.5 ^a^	12.33 ± 0.5 ^b^	11.33 ± 0.5 ^bc^	12.33 ± 0.5 ^b^	11.67 ± 0.5 ^bc^	10.67 ± 0.5 ^c^	11.33 ± 0.5 ^bc^	11.67 ± 0.5 ^bc^
*B. cereus*	20.67 ± 0.5 ^a^	8.67 ± 0.58 ^b^	8.00 ^b^	8.67 ± 0.58 ^b^	8.67 ± 0.58 ^b^	8.33 ± 0.58 ^b^	8.33 ± 0.58 ^b^	8.67 ± 0.58 ^b^
*L. monocytogene*	30.00 ± 1.0 ^a^	9.33 ± 0.58 ^b^	9.00 ^b^	9.67 ± 0.58 ^b^	10.00 ^b^	9.00 ^b^	9.00 ^b^	9.33 ± 0.58 ^b^
Gram −								
*E. coli*	23.67 ± 0.5 ^a^	9.00 ^b^	8.33 ± 0.58 ^b^	8.67 ± 0.58 ^b^	9.00 ^b^	8.67 ± 0.58 ^b^	8.67 ± 0.58 ^b^	8.33 ± 0.58 ^b^
*Sal. Typhi*	25.33 ± 0.5 ^a^	9.67 ± 0.58 ^b^	9.00 ^b^	10.00 ^b^	9.67 ± 0.058 ^b^	9.67 ± 0.58 ^b^	9.33 ± 0.58 ^b^	9.33 ± 0.58 ^b^
*P. aeruginosa*	11.00 ± 1.0 ^a^	NI ^b^	NI ^b^	NI ^b^	NI ^b^	NI ^b^	NI ^b^	NI ^b^
*E. aerogenes*	19.67 ± 0.5 ^a^	8.33 ± 0.58 ^b^	8.00 ^b^	8.33 ± 0.58 ^b^	8.00 ^b^	8.67 ± 0.58 ^b^	8.33 ± 0.58 ^b^	8.00 ^b^
Fungi								
*C. albicans*	7.08 ± 0.10 ^d^	10.68 ± 0.1 ^a–c^	9.97 ± 0.39 ^c^	11.35 ± 0.2 ^ab^	10.13 ± 0.7 ^bc^	10.42 ± 0.6 ^a–c^	11.40 ± 0.2 ^ab^	11.77 ± 0.6 ^a^

Positive Control = Tetracycline for bacteria; Nystatin for fungi, NI = No Inhibition Zone. Abbreviations are as follows—Ch: Choline chloride; Gly: Glycerol; EG: Ethylene glycol; CA: Citric acid; OA: Oxalic acid; Glu: Glucose; Fru: Fructose; W/O NADES: Without natural deep eutectic solvent. Different lowercase letters indicate significant differences based on Tukey’s pairwise comparison test (*p <* 0.05).

**Table 2:** Minimum inhibitory concentration (MIC) of EOs extracted by NADES-MAHD and without NADES.

Title 1 Bacteria	MIC (mg/mL)							
	Tetracycline	Ch:Gly 1:2	Ch:EG 1:4	Ch:CA 1:2	Ch:OA 2:1	Ch:Glu 2:1	Ch:Fru 2:1	Water (W/O NADES)
Gram +								
*S. aureus*	0.01	6.25	6.25	6.25	6.25	6.25	6.25	6.25
*S. pyogenes*	0.01	1.56	3.12	3.12	3.12	3.12	3.12	1.56
*B. cereus*	0.02	6.25	6.25	3.12	6.25	6.25	6.25	6.25
*L. monocytogene*	0.01	6.25	6.25	3.12	6.25	6.25	6.25	6.25
Gram −								
*E. coli*	0.01	6.25	6.25	6.25	6.25	6.25	6.25	6.25
*Sal. Typhi*	0.02	6.25	6.25	3.12	6.25	6.25	6.25	6.25
*P. aeruginosa*	0.06	25	12.5	12.5	12.5	25	25	25
*E. aerogenes*	0.02	6.25	6.25	6.25	6.25	6.25	6.25	6.25

Abbreviations are as follows—Ch: Choline chloride; Gly: Glycerol; EG: Ethylene glycol; CA: Citric acid; OA: Oxalic acid; Glu: Glucose; Fru: Fructose; W/O NADES: Without natural deep eutectic solvent.

**Table 3:** Antioxidant activity of EOs extracted by NADES-MAHD and without NADES.

	Ch:Gly	Ch:EG	Ch:CA	Ch:OA	Ch:Glu	Ch:Fru	Water
	1:2	1:4	1:2	2:1	2:1	2:1	W/O NADES
% DPPH Radical Scavenging	72.41 ± 1.18 ^a^	66.63 ± 1.34 ^ab^	60.81 ± 0.71 ^b^	65.95 ± 0.95 ^b^	65.67 ± 3.16 ^b^	66.06 ± 2.48 ^b^	66.51 ± 1.24 ^bc^

Abbreviations are as follows—Ch: Choline chloride; Gly: Glycerol; EG: Ethylene glycol; CA: Citric acid; OA: Oxalic acid; Glu: Glucose; Fru: Fructose; W/O NADES: Without natural deep eutectic solvent. Different lowercase letters indicate significant differences based on Tukey’s pairwise comparison test (*p <* 0.05).

**Table 4:** Assignment compounds identified in EOs of *F. vulgare* using GC-MS.

Peak No.	RT ^a^	Assignment Compounds ^b^	RI ^c^	RI Lit. ^d^
1	7.472	*m*-Cymene	1023	1023
2	7.603	D-Limonene	1027	1030
3	7.686	Eucalyptol	1030	1032
4	7.888	*trans-*β-Ocimene	1036	1049
5	8.596	γ-Terpinene	1057	1060
6	9.690	Fenchone	1089	1096
7	11.735	(-)-Camphor	1143	1142
8	13.608	α-Terpineol	1189	1189
9	14.042	Estragole	1201	1196
10	15.350	Fenchyl acetate	1233	1223
11	15.612	Cuminal	1239	1239
12	16.183	*cis*-Anethole	1253	1252
13	16.313	*p*-Anisaldehyde	1256	1270
14	18.008	*trans*-Anethole	1297	1286
15	21.611	1-(4-Methoxyphenyl)-2-propanone	1384	1384

^a^ RT refers to the retention time, expressed in minutes. ^b^ Compound identification was achieved by matching the mass spectra with reference spectra in the NIST library. ^c^ Linear retention indices (RI) were determined relative to the retention times of a homologous series of C_9_–C_26_ n-alkanes on an HP-5MS column. ^d^ RI values were obtained from the NIST Chemistry WebBook.

**Table 5:** List of NADESs for pretreatment process.

Code	Type of NADES	Molar Ratio	Code	Type of NADES	Molar Ratio
ND1	Ch:Gly	1:1	ND10	Ch:OA	3:1
ND2	Ch:Gly	1:2	ND11	Ch:OA	2:1
ND3	Ch:Gly	1:3	ND12	Ch:OA	1:1
ND4	Ch:EG	1:2	ND13	Ch:Glu	3:1
ND5	Ch:EG	1:3	ND14	Ch:Glu	2:1
ND6	Ch:EG	1:4	ND15	Ch:Glu	1:1
ND7	Ch:CA	2:1	ND16	Ch:Fru	2:1
ND8	Ch:CA	1:1	ND17	Ch:Fru	1:1
ND9	Ch:CA	1:2	ND18	Ch:Fru	1:2

## Data Availability

Data are contained within the article.

## Abbreviations

The following abbreviations are used in this manuscript:

NADES	Natural deep eutectic solvent
MAHD	Microwave-assisted hydrodistillation
HD	Hydrodistillation
EOs	Essential oils
S/N	Solid-to-NADES
Ch	Choline chloride
Gly	Glycerol
EG	Ethylene glycol
CA	Citric acid
OA	Oxalic acid
Glu	Glucose
Fru	Fructose
MHs	Monoterpene hydrocarbons
OMs	Oxygenated monoterpenes
PPs	Phenylpropanoids
